# Early Childhood Lower Respiratory Illness and Air Pollution

**DOI:** 10.1289/ehp.9617

**Published:** 2007-05-22

**Authors:** Irva Hertz-Picciotto, Rebecca James Baker, Poh-Sin Yap, Miroslav Dostál, Jesse P. Joad, Michael Lipsett, Teri Greenfield, Caroline E.W. Herr, Ivan Beneš, Robert H. Shumway, Kent E. Pinkerton, Radim Šrám

**Affiliations:** 1 Department of Public Health Sciences, University of California, Davis, California, USA; 2 Department of Epidemiology, University of North Carolina, Chapel Hill, North Carolina, USA; 3 Laboratory of Genetic Ecotoxicology, Institute of Experimental Medicine, AS CR and Health Institute of Central Bohemia, Prague, Czech Republic; 4 Department of Pediatrics, University of California, Davis, California, USA; 5 Department of Epidemiology and Biostatistics, University of California, San Francisco, California, USA; 6 Institute of Hygiene and Environmental Medicine, University of Giessen, Giessen, Germany; 7 Health Institute Usti n.L., Branch Teplice, Czech Republic; 8 Department of Statistics, University of California, Davis, California, USA; 9 Department of Rheumatology, Allergy and Clinical Immunology, University of California, Davis, California, USA

**Keywords:** air pollution, bronchitis, children’s health, infant, particulate matter, PM_2.5_, PAHs, polycyclic aromatic hydrocarbons, respiratory illness, volatile organic compounds

## Abstract

**Background:**

Few studies of air pollutants address morbidity in preschool children. In this study we evaluated bronchitis in children from two Czech districts: Teplice, with high ambient air pollution, and Prachatice, characterized by lower exposures.

**Objectives:**

Our goal was to examine rates of lower respiratory illnesses in preschool children in relation to ambient particles and hydrocarbons.

**Methods:**

Air monitoring for particulate matter < 2.5 μm in diameter (PM_2.5_) and polycyclic aromatic hydrocarbons (PAHs) was conducted daily, every third day, or every sixth day. Children born May 1994 through December 1998 were followed to 3 or 4.5 years of age to ascertain illness diagnoses. Mothers completed questionnaires at birth and at follow-up regarding demographic, lifestyle, reproductive, and home environmental factors. Longitudinal multivariate repeated-measures analysis was used to quantify rate ratios for bronchitis and for total lower respiratory illnesses in 1,133 children.

**Results:**

After adjustment for season, temperature, and other covariates, bronchitis rates increased with rising pollutant concentrations. Below 2 years of age, increments in 30-day averages of 100 ng/m^3^ PAHs and of 25 μg/m^3^ PM_2.5_ resulted in rate ratios (RRs) for bronchitis of 1.29 [95 % confidence interval (CI), 1.07–1.54] and 1.30 (95% CI, 1.08–1.58), respectively; from 2 to 4.5 years of age, these RRs were 1.56 (95% CI, 1.22–2.00) and 1.23 (95% CI, 0.94–1.62), respectively.

**Conclusion:**

Ambient PAHs and fine particles were associated with early-life susceptibility to bronchitis. Associations were stronger for longer pollutant-averaging periods and, among children > 2 years of age, for PAHs compared with fine particles. Preschool-age children may be particularly vulnerable to air pollution–induced illnesses.

Research linking air pollution with morbidity and mortality indicates the strongest effects on the very young and the elderly. Higher infant and early childhood mortality has been associated with elevated ambient particle concentrations in Brazil (Penna and Duchiade 1991), Taiwan (Knobel et al. 1995), the Czech Republic (Bobak and Leon 1999), the United States (Woodruff et al. 1997), and Mexico (Loomis et al. 1999). A recent review suggests that the most consistent associations have been for respiratory causes of death in the post-neonatal period (Glinianaia et al. 2004a). In older, mostly school-age children, ambient air pollutants have been associated with daily hospital admissions, reduced lung function, reported respiratory symptoms, and increased use of asthma medication (Millstein et al. 2004; Pope et al. 1991; van der Zee et al. 1999).

Although the first few years of life are considered an especially vulnerable period, few studies have examined air pollution in relation to infant and early childhood morbidity. In Chile, Ostro et al. (1999) found particulate matter < 10 μm in aerodynamic diameter (PM_10_) to be associated with elevated daily counts of emergency room visits for lower respiratory symptoms among children < 2 years of age. Others (Farrow et al. 1997; Samet et al. 1993) observed no association between indoor nitrogen dioxide concentrations and incidence or severity of respiratory illness among infants.

Many constituents of ambient air pollution from manufacturing, motor vehicles, and home heating are also components of cigarette smoke, including PM and many polycyclic aromatic hydrocarbons (PAHs). Exposure to environmental tobacco smoke (ETS) places children at greater risk for low birth weight, perinatal mortality, deficits in childhood growth, sudden infant death syndrome, middle ear disease, bronchitis, pneumonia, cough, asthma, and wheeze (DiFranza and Lew 1995, 1996; Fox et al. 1990; Strachan and Cook 1997). DNA and hemoglobin adducts and chromosomal aberrations are increased by transplacental ETS exposure (Coghlin et al. 1991; Hansen et al. 1992).

Given the sparse literature on morbidity in infants and preschool-age children, a birth cohort study was launched in 1994 in two districts in the Czech Republic as part of the Teplice program of research on exposure, bio-markers, and health effects of ambient pollution (Šrám et al. 1996). Teplice is a coal mining district with numerous large power plants that historically supplied energy to much of the former Czechoslovakia; it was known for its high levels of air pollution. The other district, Prachatice, is characterized by light industry and lower levels of particulate air pollution. We used data from an intensive long-term air pollution monitoring program in both districts to examine whether short-term exposures to ambient particulate matter < 2.5 μm in aerodynamic diameter (PM_2.5_) and PAHs would increase the risk for childhood respiratory illnesses in the preschool period, after adjusting for household and other covariates.

## Methods

### Enrollment and data collection

From May 1994 through March 1999, about 90% (*n* = 7,502) of women who delivered in the districts of Teplice or Prachatice participated in the Pregnancy Outcome Study (Dejmek et al. 2000). While in the hospital, mothers completed questionnaires on work history, demographics, lifestyle, and reproductive and medical histories.

A stratified random sample of 1,492 mother–infant pairs from the Pregnancy Outcome Study was recruited into the Immune Biomarker Study (IBS) (Hertz-Picciotto et al. 2002, 2005). Data on pregnancy, labor, delivery, and the neonate were abstracted from the medical records of IBS participants, including birth weight, length of gestation, maternal hypertension and diabetes, and infant APGAR score. Sampling of low birth weight and preterm births was at a higher fraction than that of normal full-term infants. Sampling fractions increased in later years. The overall sampling fraction was 20%.

The Czech Early Childhood Health (CzECH) study was a longitudinal follow-up of the IBS births. Children born 1994–1996 were followed up at 3 years of age, and those born 1997–1998 were followed up at 4.5 years of age. Thus, this cohort study followed up each child once to obtain medical record and home environmental information.

Pediatricians and nurses identified the selected children in their practices, administered informed consent, distributed parental questionnaires, and abstracted the medical records. The use of a uniform pediatric medical records form throughout the country facilitated collection of *International Classification of Diseases, Tenth Revision* (ICD-10; World Health Organization 1993) codes for all diagnoses during physician visits or hospitalizations. Czech physicians assign ICD codes as part of their regular practice. Data on all hospitalizations and visits to specialists are forwarded to the primary physician, who in this case was the pediatrician with whom the child was registered. In the Czech Republic, each child is registered with a pediatrician. Participation by pediatricians was 100%.

We conducted a validation study to determine how diagnoses of bronchitis and croup are made in the two districts and to assess consistency across practices and between the two districts. Twenty-five pediatricians answered seven questions about their coding of specific symptoms and use of specific ICD codes for various lower respiratory illnesses (survey available on request).

The parental questionnaire asked about the child’s early environment: breast-feeding; day care or preschool attendance; type of building construction for the home; home heating fuel; device and fuel used for cooking; ages and smoking status of all household members; and so forth. Forms were developed in Czech, translated into English, revised, and back-translated. This study complied with all applicable U.S. and international requirements and was approved by the institutional review boards of the Regional Institute of Hygiene of Central Bohemia, Prague; the University of North Carolina, Chapel Hill; and the University of California, Davis, School of Medicine. All participants gave written informed consent before data collection.

### Respiratory illnesses

We focused on lower respiratory illnesses (LRI) based on ICD-10 codes. The vast majority were acute laryngitis and tracheitis (ICD-10 code J04) and acute bronchitis (J20). We assessed two subsets:

First, croup was defined as acute infectious illness with bark-like cough and inspiratory stridor. This category comprised acute laryngitis and tracheitis (J04, *n* = 1,580) and acute obstructive laryngitis (croup) and epiglottitis (J05, *n* = 2). Although the subglottic space, which is the area of narrowing responsible for inspiratory stridor and the seal-like barking quality of the cough, could be considered part of either the upper or lower airway, for consistency with studies such as the Tucson Children’s Respiratory Study (Taussig et al. 1989) and the Multicentre Allergy Study Group (Illi et al. 2001), we included croup with LRI.

Second, bronchitis/bronchiolitis was defined as acute illness with lower airway sounds such as wheeze and rhonchi. This category comprised acute bronchitis (J20, *n* = 2,566) and acute bronchiolitis (J21, *n* = 1). Responses to the pediatrician survey indicated that the distinction typically made in the United States between bronchitis and bronchiolitis based on age of diagnosis is not used in the Czech Republic.

Third, an overall category of LRI was defined as any of the above diagnoses plus other chronic obstructive pulmonary disease (COPD) (J44, *n* = 39), pneumonia (J12, J14, J15, J16, and J18, *n* = 151), and asthma (J45, *n* = 47). Because of small numbers, separate analyses were not conducted for COPD, pneumonia, or asthma.

### Exposure assessment

In January 1992, the Czech Ministry of Environment, the Czech Institute of Hygiene, and the U.S. Environmental Protection Agency (Pinto et al. 1998) initiated an air monitoring program with sites in Teplice and Prachatice. Measurements of PM_2.5_, PM_10_, and PAHs were performed daily in November–March, every third day in April–June and September–October, and every sixth day in July–August. Sulfur dioxide, oxides of nitrogen, nitric oxide, NO_2_, and ozone were measured year-round on a daily basis and were used, along with PAH and particle data, in imputation for days without scheduled measurements of these latter two. The assumed imputation model related the current log-transformed pollution vector to an underlying vector pollution signal, with components consisting of SO_2_, PM_10_, NO_x_, PM_2.5_, and PAH. The underlying pollution signal was assumed to be a first-order vector autoregressive process. Imputed values are the conditional expectations for the missing data values, conditioned on past values of the series itself and current and past values of the other series. Final imputed values are the exponentially transformed values of the imputed logarithmic values (Hertz-Picciotto et al. 2005; Little and Rubin 2002; Shumway and Stoffer 2000).

Pollutants were measured in samples collected by the Versatile Air Pollution Sampler (VAPS) device (Pinto et al. 1998). Air is drawn through the VAPS inlet, which has a limit of 10 μm. A virtual impactor separates the airflow into two channels that collect fine particles (< 2.5 μm) and a third that collects coarse particles (2.5–10 μm). Teflon filters collect the fine and coarse particles, with mass determined gravimetrically using microbalances, which undergo annual certification by the Czech Metrological Institute. Quality assurance and quality control protocols were modeled after those of the U.S. Environmental Protection Agency (1989).

In the second fine-particle channel, a 25 × 100 mm polyurethane foam (PUF) trap located downstream of a 47-mm quartz filter collected gas-phase PAHs. Extraction from both the PUF trap and the quartz filters was followed by high-performance liquid chromatography analysis with a fluorescence detector and UV detector. Twelve PAHs were measured in both the gas and particle phases and summed to create “total PAHs”: phenanthrene, anthracene, fluoranthene, pyrene, benzo[*a*]anthracene, chrysene, benzo[*b*]fluoranthene, benzo[*k*]fluoranthene, benzo[*a*]pyrene, dibenzo[*ah*]anthracene, benzo[*ghi*]perylene, and indeno[1,2,3-*cd*]pyrene. Missing values for specific PAH compounds were imputed following standard procedures (Little and Rubin 2002).

Elimination of interference was accomplished by use of blanks, decontamination of all laboratory glassware using appropriate solvents, and repurification of the extract before the analysis. External standards were supplied by Dr. Ehrenstorfer GmbH (Augsberg, Germany). Calibration was carried out at least once per week with at least five concentration levels within the range of 5–1,000 ng/mL using a linear response to analyte regression.

### Statistical methods and data analysis

#### Data management and preparation

Electronic data entry took place in Prague. A secure Web-based data entry system designed at the University of California, Davis, was used for the follow-up study. A 10% audit indicated fewer than four entry errors per thousand fields. Statistical programmers in the United States conducted extensive cleaning: Outliers, implausible values, missing data, and inconsistencies were checked against hard copies and, where necessary, one of us (M.D.) re-reviewed medical records or re-contacted parents or physicians.

#### Identification of confounders

A three-pronged strategy for identification of confounders involved review of the literature; development of a causal diagram (directed acyclic graph; DAG) using established or hypothesized associations among relevant variables (Hernan et al. 2002); and empirical stratified analyses. Rates per child-month of LRI/bronchitis/croup were calculated overall and within strata of covariates, and rate ratios were determined. We also examined associations of covariates with air pollution, and those that met criteria for potential confounding or were predictors that, once controlled, did not open up a backdoor path on the DAG were retained for inclusion in the initial full multivariable models.

#### Multivariate analyses

To quantify associations between air pollutant exposures and early childhood respiratory morbidity, we fit generalized linear longitudinal models using the logit link and binomial errors. The data set was structured with each observation representing one child-day. Columns for time-dependent variables were exposure (e.g., average 30-day PAH exposure), illness event indicators, changing covariates (e.g., age of child, current breast-feeding status, ETS), and calendar factors (season, day of week). Time-invariant child-specific covariates were also included (e.g., year of birth, sex).

The date of diagnosis in the medical chart served as a proxy for the time of illness occurrence. Because parents do not generally report back to the pediatrician when the child recovers, the duration of illness is unknown. To ensure that only incident events were analyzed, identical diagnoses within 1 month for the same child were considered the same illness, and the 29 days after the date of initial diagnosis were therefore filtered out. Pediatricians’ diagnoses recorded separated by ≥ 30 days were treated as separate events.

For each outcome, we fitted a full model that included PAH concentrations and potential confounders, after eliminating or combining redundant or collinear variables. Subsequently, we removed variables that did not predict the outcome with adequate precision (*p* > 0.15) and were not confounders (removal resulted in changes < 15% in the estimated coefficient for PAHs). Once this set of predictive covariates was determined, the same variables were retained for PM_2.5_ models and in all sensitivity analyses. Only single-pollutant models were fit.

We used generalized estimating equations to adjust for within-subject correlations arising from repeated days of observation (Hertz-Picciotto et al. 2000; Zeger et al. 1988) and evaluated three covariance structures: independent, autoregressive, and exchangeable. The coefficients were essentially unchanged, and because the exchangeable covariance matrix resulted in slightly smaller standard errors, it was used in all further models. If some children are genetically predisposed or at higher risk due to other unmeasured but relatively stable aspects of their immediate environment, then the exchangeable covariance is supported for biological reasons. Robust variance estimates were obtained.

We calculated the average 3-day air pollutant concentrations using the same day and two previous days, and similarly for the 7-, 14-, 30-, and 45-day averages. We used the same averaging periods for temperature. Long-term time trends were adjusted using a linear term, because nonlinearity was not detected. To evaluate model fit, we calculated Akaike Information Criterion (AIC) statistics (Akaike 1973). All models were fit using SUDAAN statistical software version 8 (http://www.rti.org/sudaan) with adjustment for the sampling design: stratified sampling without replacement in strata defined by district, year of birth, and preterm or low-birth-weight status. Inverse probability sampling weights were used.

Odds ratios were estimated from the logistic model for a fixed increase in concentration of PAHs (100 ng/m^3^) or of PM_2.5_ (25 μg/m^3^). These values are close to two standard deviations of the respective pollutant distributions over the entire study period. Hence, the reported rate ratios are comparable in this population for these two pollutants. Given the low probability of an illness on a given day of life for a given child (0.003 for all LRI), the odds ratios [exp( β × g)] closely approximate the rate ratios.

The effects of child’s age and of breast-feeding differed in children below versus above two years of age. To simplify presentation, we constructed separate models for birth through 23 months and for 2 to 4.5 years of age. We conducted sensitivity analyses using the months with daily air pollutant monitoring only, and by restricting the analysis to subjects for whom pollutant concentrations at their residences would correspond closely to measurements at the fixed-site monitors. Rather than a simple distance measure, one of the authors (I.B.) used knowledge of the landscape and his expertise in air pollution monitoring to assign levels of likely concordance between each household and the monitors.

## Results

### Air pollution

**Figure 1:**
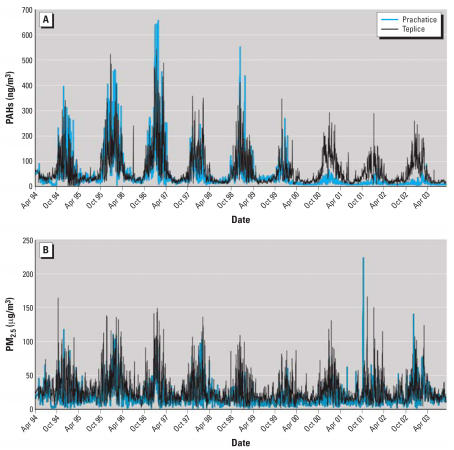
Time series for daily PAHs (*A*) and PM_2.5_ (*B*) in two districts of the Czech Republic, May 1994–August 2003.

Concentrations of PAHs and PM_2.5_ peaked in winter months (Figure 1). PM_2.5_ concentrations were generally higher in Teplice than in Prachatice, but PAHs were similar until 1999. The mean daily PAH concentration during the study period was 52.5 ng/m^3^ and the mean daily PM_2.5_ concentration was 22.3 μg/m^3^. On 10% of days, PAHs exceeded 146 ng/m^3^ in Teplice and 104 ng/m^3^ in Prachatice, and PM_2.5_ exceeded 52 μg/m^3^ and 35 μg/m^3^ in the two districts, respectively.

Standard deviations of PAHs varied from 57 ng/m^3^ (for the 3-day average) to 46 ng/m^3^ (45-day average), and of PM_2.5_ from 16 to 11 μg/m^3^. Thus, the benchmark increments we used (100 ng/m^3^ PAHs, 25 μg/m^3^ PM_2.5_) are roughly two times the standard deviations of the air pollutant distributions. The low variability during summer months in air pollution, especially PAH exposures (Figure 1) implies that the error introduced by imputation is likely to have been small.

Temperature averaged over 14 days showed a strong negative correlation with 30-day averages of both PAHs (–0.86 in Teplice, –0.53 in Prachatice) and PM_2.5_ (–0.68 in Teplice, –0.58 in Prachatice).

### Follow-up

Of 1,265 eligible families for whom contact was attempted, response rates were 95% and 97% for the 1994–1996 births and 1997–1998 births, respectively, for a combined group of 1,133 children with complete data (Table 1). Differences between the original Pregnancy Outcome Study cohort and these 1,133 in the follow-up study were primarily related to the *a priori* sampling design, based on variables such as year of birth and district of residence; otherwise, demographic, lifestyle, and newborn characteristics were generally similar, although mothers of low parity were slightly more likely to participate, and Roma mothers slightly less (Table 2).

Based on information collected at follow-up, 35% of mothers smoked at some point after delivery, and in about half the households another adult smoked. Coal was a fuel source in 10% of households. About 88% of the children breast-fed: 50% for ≥ 4 months and 10% for > 1 year. Close to 60% lived in a household with at least one other child. At 3 years, one-fifth had ever attended day care or nursery school, compared with four-fifths of those followed to 4.5 years of age.

### Respiratory illness rates

The overall rates for lower respiratory illness, bronchitis, and croup in children < 2 years of age were 83, 55, and 27 per 1,000 child-months, respectively (or expressed equivalently, 8.3%, 5.5% and 2.7% per month). Among those ≥ 24 months of age, the rates were 68, 38, and 28 per 1,000 child-months. Notably, a sizable proportion of children contributed multiple events: > 250 experienced four or more episodes of LRI separated by ≥ 30 days within the first 3 years of life. Because more than half of the LRI events were bronchitis, we focused the presentation of results on the latter.

### Bivariate prediction of respiratory illness

In bivariate analyses in children < 2 years of age, high average PAH exposure for the previous 30 days (> 100 ng/m^3^) was associated with bronchitis rates that were more than double those of low exposure periods (previous month’s average PAH < 40 ng/m^3^) (Table 3). Risk was similarly increased after high PM_2.5_ exposure (30-day average > 50 vs. < 25 μg/m^3^). Bronchitis rates in this age group were also higher in boys, children of mothers with lower education, and children from homes with adults who smoke, or homes in which coal was used for heating or cooking. Current or recent breast-feeding was protective, as was older maternal age.

In children 2–4.5 years of age, patterns were similar for air pollutants and most other factors, but attenuated for smoking in the household, use of coal for heating/cooking, ethnicity, and maternal education (Table 4). In 2- to 4.5-year-olds, day care or preschool attendance conferred a 47% increase in bronchitis rates [rate ratio (RR) = 1.47; 95% confidence interval (CI), 1.29–1.66].

### Multivariate adjusted results

**Figure 2:**
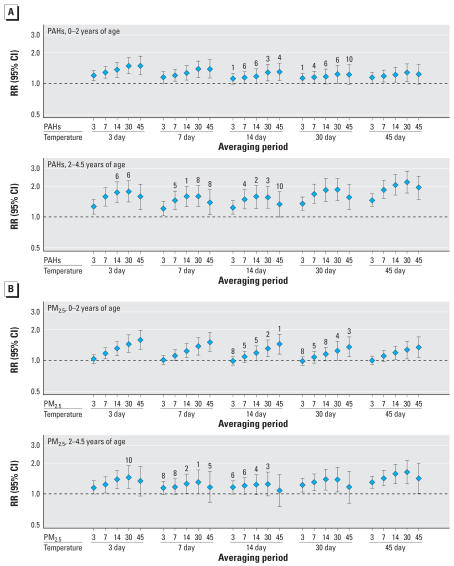
Bronchitis RRs and 95% CIs for two air pollutant classes: (*A*) PAHs and (*B*) PM_2.5_ , for children 0–2 years of age (upper panel for each pollutant) and 2–4.5 years of age (lower panel for each pollutant). In each set of panels, RRs for five averaging periods for the pollutant are presented, with adjustment for each of five averaging periods for mean daily temperature. Rank ordering by goodness of fit (1 = best), calculated from the AIC, is shown by the numerals above the 10 best-fitting models in each panel. Fit statistics spanned a rather tight range. Notably, the strongest associations were not necessarily from the best-fitting models.

In multivariable models, elevated rates of bronchitis in infants and toddlers were observed with higher PAH exposures, especially for longer averaging periods of 30 or 45 days (Figure 2). After adjustment for 14-day average temperature and other covariates, an incremental increase in average 30-day PAH exposure of 100 ng/m^3^ was associated with an RR for bronchitis from birth to 2 years of 1.29 (95% CI, 1.07–1.54) (Table 3). Adjusted for 3-day average temperature, the RR was 1.49 (95% CI, 1.24–1.80). Rates decreased on weekends and increased on Mondays over other weekdays; rates were elevated in fall, winter, and spring, compared with summer.

PAH associations with bronchitis were especially strong in the older age group (2–4.5 years) for all averaging periods of 7, 14, or 30 days (Figure 2). The 30-day PAH exposure increment was associated with a multivariate adjusted RR of 1.56 (95% CI, 1.22–2.00) adjusted for 14-day temperature (Table 4), and an RR of 1.77 (95% CI, 1.40–2.25) adjusted for 3-day temperature. The magnitude of the increased risk varied from a low of 1.21 (3-day average PAHs adjusted for 7-day average temperature) to a high of 2.20 (30-day average PAHs adjusted for 45-day temperature) depending on choice of averaging periods, but the finding of elevated bronchitis rates after high PAH exposures was robust. Based on the AIC, using 14-day average temperature and 30-day average PAHs always resulted in one of the three best-fitting models, regardless of age group or air pollutant, though the differences in AIC among the best five models were generally very small.

For PM_2.5_, the 30-day increment of 25 μg/m^3^, after adjustment for 14-day temperature, conferred an RR of 1.30 (95% CI, 1.08–1.58) between birth and 2 years of age. Generally, however, RRs tended to be lower than for the two standard deviation increments of PAHs and were statistically significant less often. From birth to 2 years, the strongest PM_2.5_ effects occurred for 14-, 30-, or 45-day averages, especially when the temperature averaging period was short. Above 2 years of age, although all point estimates were positive, significant results for fine particles occurred mostly with adjustment for the longest or shortest temperature averaging periods (3, 30, or 45 days); the highest rate ratios in this age group were for 30-day average PM_2.5_, similar to the results for PAHs.

Because bronchitis represented most LRI events, associations with the broader LRI category were similar to or slightly lower than those for bronchitis alone (results available on request). Despite strong associations of croup with PAHs in bivariate analyses of children < 2 years of age, multivariable-adjusted models showed no consistent pattern in relation to the air pollutants examined in either age category (results available on request).

To evaluate the potential impact of errors introduced through imputation, we conducted analyses for November–March only, when PM_2.5_ and PAHs were measured daily. In the older preschool children, the RR for 30-day PAHs increased markedly from 1.56 (95% CI, 1.22–2.00) in the year-round model to 1.75 (95% CI, 1.28–2.40) in the model based on periods with daily monitoring, whereas the nonsignificant result for 30-day PM_2.5_ remained nonsignificant (RR = 1.23, 95% CI, 0.94–1.62) in the year-round model compared with RR = 1.17 (95% CI, 0.85–1.60) in the 5-months-per-year model). In the younger group, changes in RRs were small (from 1.29 to 1.16 for PAHs, and 1.30 to 1.23 for PM_2.5_), possibly reflecting less time spent outdoors. Sensitivity analyses restricted to residences with the greatest probability that air pollution exposures are similar to measurements at the monitoring site again showed stronger effects in the 2- to 4.5-year olds for PAHs (RR = 1.74; 95% CI, 1.10–2.76) and for PM_2.5_ (RR = 1.33; 95% CI, 0.85–2.10), though the latter remained nonsignificant. Removal of covariates one by one, except temperature, altered the air pollutant RRs by < 20%.

## Discussion

This birth cohort study had daily air pollutant data for 5 months each year, respiratory outcomes over a 10-year calendar period, and individual-level time-varying home environmental factors on > 1,000 children. It is the first study to relate a large database of ambient PAH measurements to respiratory disease.

Early childhood respiratory illnesses account for much of the morbidity in the youngest segments of the population. LRIs are more serious than illnesses affecting upper airways, often resulting in lost workdays for employed parents. In this study, LRI incidence was 8.3 per 1,000 child-months, more than one event per year per child, on average.

Our major finding is a clear demonstration that PAHs were associated with a greater incidence of physician-diagnosed LRIs, particularly bronchitis, in preschool children, even after adjustment for temperature, season, calendar time trends, and multiple individual characteristics. The strongest associations were observed in preschool children ≥ 2 years of age; this group may have been either more susceptible or more highly exposed for a given ambient level. Exposures from ambient air pollution sources might be greater in the older children if infants and young toddlers were kept indoors more, especially in winter months when pollutant levels are higher. Associations of PM_2.5_ with bronchitis in this age group were weaker and less consistent than for PAHs. Croup was not associated with these pollutants after adjustment for confounders.

Preschoolers > 1 year of age have been studied very little: Research based on parental reports of symptoms showed elevated rates of cough without a cold and wheeze in association with PM_10_ (Pierse et al. 2006), and of ear, nose, and throat infections in association with higher PM_2.5_ (Brauer et al. 2002). A study similar to ours (Pino et al. 2004) used physician diagnoses of Chilean infants from 4 months to 1 year of age and reported that an average 10-μg/m^3^ increase in fine particles, lagged 9 days, was associated with a 9% increased risk of wheezing bronchitis.

We observed a similar increase of 7% for all bronchitis when we calculated the RR for a 10-μg/m^3^ increment averaged over 14 days with no lag; our results were stronger for longer averaging periods (≥ 30 days), which Pino and colleagues did not examine. We are aware of only a few other investigations of children in which measurements of PAHs were obtained. Miller et al. (2004) followed pregnant women in New York City who wore personal monitors for 48 hr during the third trimester, and found that maternally reported respiratory symptoms during the first 2 years of life increased with PAHs among those children who were also postnatally exposed to environmental tobacco smoke. Using a similar study design in Poland, with monitoring during the second trimester, researchers observed high relative risks for maternally reported barking cough, wheezing without cold, and other symptoms, as well as longer duration of respiratory symptoms (Jedrychowski et al. 2005).

Hospital admissions (Gouveia and Fletcher 2000) and mortality (Loomis et al. 1999) in the first year of life are significantly increased after episodes of high air pollution. Post-neonates were the most vulnerable to total and respiratory mortality in a Korean study of PM_10_ (Ha et al. 2001). Two studies of linked birth–infant death files also found that post-neonatal mortality from respiratory illness was increased by high exposures to ambient particles: Ritz et al. (2006) examined average concentrations of PM_10_ for periods of 2 weeks to 6 months before deaths of infants up to 12 months of age; Woodruff and colleagues (2006) analyzed infants’ lifetime average exposure to PM_2.5_. Both studies observed a doubling of postneonatal mortality in relation to particulate matter concentrations. A systematic review of 15 studies of infant mortality and air pollution concluded that results are most consistent for respiratory deaths in postneonates (Glinianaia et al. 2004a). No overall association was observed between hospitalizations of infants with bronchiolitis, primarily from respiratory syncytial virus, and acute exposures to PM_2.5_, but elevated risks were found for lags of 3–5 or 6–8 days among those born at < 29 weeks gestation (Karr et al. 2006). However, subchronic exposures, defined as exposures in the month preceding hospitalization, were associated with higher risks for bronchiolitis in the first year of life: An increase in PM_2.5_ of 10 μg/m^3^ was associated with a relative risk of 1.09 (95% CI, 1.04–1.14) (Karr et al. 2007). Interestingly, if converted to the increment used in our analyses, namely 25 μg/m^3^, the relative risk for PM_2.5_ is 1.24, quite similar to the 1.30 that we obtained for the first 2 years of life.

Temperature and air pollution are correlated with each other, and both are associated with lower respiratory illness. Regardless of the temperature adjustment, the association of PAH exposures with bronchitis was strongest for the 30-day pollutant average. PAHs were significant in all 25 models fit to the data on 2- to 4.5-year-olds, and in 21 of 25 models in the younger age group. In contrast, associations of PM_2.5_ with bronchitis were significant primarily for 30- and 45-day averages in the younger age group, and for 3-to 30-day averages in 2- to 4.5-year-olds, after adjustment for long averaging periods of temperature.

We defined illness events using ICD-coded physician diagnoses. Thus, the event must impel the parent to bring the child to a physician, who must then make a correct diagnosis. All Czech citizens are entitled to free, readily available medical care. Families usually remain with one pediatrician. We attribute the low refusal rate in the follow-up study (5.4% for births in 1994–1996, without incentives, and 2.5% in 1997–1998 births, when incentives were offered) to the close relationships between the family and the physician and nurses. Ready access to and high utilization of physicians are demonstrated by the completeness of immunizations: 98% of the children received a complete series of four DPT (diphtheria–pertussis–tetanus) injections, compared with 81% of U.S. children in 1997 (Centers for Disease Control and Prevention 1999).

Studies of child morbidity often rely on parental reports, usually collected retrospectively, which can be inaccurate and highly subjective (Lara et al. 1998). In contrast, the validation survey we conducted with 25 pediatricians indicated strong consistency in coding symptoms of bronchitis and croup, and no differences between districts (survey instrument and results available on request). Whatever their limitations, physician diagnoses are recorded at the time of the consultation and are more objective than parental reports and more complete than hospitalizations alone. Moreover, because visits to specialists or hospitals are forwarded to the primary physician in the Czech Republic, diagnoses in this study included virtually all contacts with health care providers.

We focused on episodes of LRI, which are more likely to result in contact with the health care system than, for instance, the occurrence of less serious illness, such as an upper respiratory infection. However, because our primary air pollution comparisons are temporal, not spatial, variation in health care utilization or diagnostic practices (Howel et al. 2001) is less likely to be associated with pollution and hence would not result in confounding. We also assessed possible shifts in diagnostic practices or health care–seeking behavior (results available on request), but found little evidence for time trends or differences across districts. Had they existed, statistical adjustment for calendar time and district would have controlled for them. We did observe that children born in 1995 or 1996 appeared to experience higher illness rates, respiratory and nonrespiratory, before 2 years of age. Because both years were characterized by particularly high levels of pollution, peri-natal exposures may have influenced the health of these birth cohorts.

Concentrations of organic pollutants and particles in this study were, as previously reported (Hertz-Picciotto et al. 2005), comparable to those recorded in a number of U.S., European, and Asian cities (Naumova et al. 2002; Pinto et al. 2004). This similarity in ambient air pollutant levels supports generalizability of our findings. Moreover, whereas many air pollution studies have measurements only every sixth day, we obtained daily data on PM_2.5_ and on both gaseous and particle-bound PAHs for 5 months each year for 10 years, and every-third-day measurements for another 5 months per year. Major findings were similar or stronger in analyses of months with daily data only. Availability of frequent measurements permitted accurate differentiation of effects for different averaging periods.

We chose to examine fine rather than coarse particles because, with only one monitor in each district, exposure misclassification error would likely be lower. The striking findings for PAHs but not for PM_2.5_ are unlikely to be an artifact; when we limited analyses to children residing at closer distances or not separated from monitors by topography, the patterns were similar: The RR for 30-day PAHs increased from 1.56 to 1.74 in children > 2 years of age, and the nonsignificant findings for PM_2.5_ remained so.

Potential mechanisms by which PAHs or PM_2.5_ may increase LRIs are numerous, including oxidative stress, structural damage, efficient transport of pathogenic microbes, and immune dysregulation. Oxidative stress is strongly correlated with organic carbon components, specifically PAHs (Li et al. 2003). PAH constituents of diesel exhaust particles catalytically generate reactive oxygen species, causing stress to biological systems (Hiura et al. 1999). Several metabolic and cellular activation pathways appear linked to PAHs, and may affect cytokine and chemokine expression. (D’Arena et al. 1998). Particles can also impair alveolar macrophage superoxide production (Kleinman et al. 2003), which may in turn compromise the lung’s ability to kill some respiratory pathogens. Pathways involving immunologic alterations are supported by our previous finding that PM_2.5_ exposures during the 14 days before delivery were associated with reduced T-lymphocyte percentages and elevated B-lymphocyte percentages (Hertz-Picciotto et al. 2005).

Despite strong biological plausibility, our results cannot be presumed to represent causal associations without further investigation of the roles of other pollutants, such as O_3_, PM_10_, carbon monoxide, NO_2_, and metals, which have been associated with a variety of respiratory diagnoses (Fusco et al. 2001; Gehring et al. 2002; Hruba et al. 2001; Ilabaca 1999; Lipsett et al. 1997).

Sensitivity analyses exploring different averaging periods for pollutants and temperature, different covariance assumptions, the impact of imputation, and so forth, yielded consistent patterns of results. Such robustness of the principal results to analytic decisions strengthens the plausibility of a causal link. Overall data validity was supported by confirmation of established risk and protective factors (e.g., current breast-feeding, presence of other children in the household, low maternal education, child’s sex, and ETS exposure) (Koch et al. 2003; Pino et al. 2004).

To summarize, this study demonstrated strong associations of PAHs with lower respiratory illnesses, especially bronchitis, in children between birth and 4.5 years of age. These associations are unlikely to have been confounded, subject to the caveat that we did not examine other components of ambient air pollution or meteorologic covariates besides temperature. Strengths of the study include participation of all physicians and high retention rates, which minimized the possibility of selection bias; the high quality and intensive air monitoring program; and a wealth of covariate data that were well controlled in the statistical analysis, including breast-feeding, day care attendance, indoor sources of air pollution, ambient temperature, and season. The case for generalizability of the results, should they prove to be causal, is strong, given that the analysis accounted for sampling fractions and that exposure levels were comparable to those in cities throughout western Europe, the United States, and elsewhere. Experimental research suggests that a causal relationship with PAHs and PM_2.5_ is plausible, though our data support the former more than the latter. Whereas ambient air quality standards focus on particulate matter and gaseous pollutants such as SO_2_, CO, and O_3_, PAHs are ubiquitous, and few epidemiologic studies have examined their associations with morbidity. This study indicates that short-term exposures to PAHs may represent a significant public health threat to children.

## Correction

In “Respiratory illnesses,” some of the numbers of events under various ICD-10 codes; the rates of croup in “Respiratory illness rates”; and some values in Table 3 for “Day of the week” were incorrect in the manuscript originally published online. They have been corrected here. Also, different averaging periods are presented for correlations of temperature and air pollutants. A new paragraph on studies of PAHs has been added to the “Discussion.”

## Figures and Tables

**Table 1:** Evolution of CzECH study samples from two periods [no. (%)].

Characteristic	Births 1994–1996	Births 1997–1999
Participants in immune biomarker study at delivery (*n* = 1,492)	615	877
Exclusions		
Family moved outside of the district	32	80
Child was adopted or put into social care	11	7
Family not found	9	0
Child died	7	4
Mother died	0	4
Othera	2	3
Total ineligible	61 (10)	98 (11)
Eligible for follow-up study	554	779
Contact not attempted^b^	—	68 (8.7)
Eligible for follow-up study, contact attempted	554	711
Refused to participate in follow-up	30 (5.4)	18 (2.5)
Agreed to participate with medical record review	524	693
No maternal delivery questionnaire data	0	6 (0.8)
No maternal questionnaire at follow-up visit	72 (13.0)	6 (0.8)
Participants in the follow-up study with complete data	452 (82^c^)	681 (96^c^)

a Missing diagnosis data, seeing physician in another district, or mother mentally unstable.

b Health Effects Institute decision was that births January–March 1999 not be contacted for follow-up.

c Percent of those who were eligible and for whom contact was attempted.

**Table 2:** Characteristics of the participants in the Pregnancy Outcome Study and the CzECH follow-up study [no. (%)].^a^

Characteristic	Pregnancy Outcome Study (*n* = 7,502)	Participants in CzECH study follow-up (*n* = 1,133)
District		
Prachatice	2,144 (29)	485 (43)
Teplice	5,358 (71)	648 (57)
Total	7,502 (100)	1,133 (100)
Season of birth		
Winter	1,826 (24)	287 (25)
Spring	2,017 (27)	316 (28)
Summer	1,929 (26)	265 (23)
Autumn	1,730 (23)	265 (23)
Year of birth		
1994	1,313 (18)	76 (7)
1995	1,606 (21)	142 (13)
1996	1,420 (19)	234 (21)
1997	1,419 (19)	337 (30)
1998	1,394 (19)	344 (30)
Infant’s sex		
Male	3,856 (51)	576 (51)
Female	3,643 (49)	557 (49)
Birth weight (g)		
< 2,500	365 (5)	81 (7)
≥ 2,500	7,132 (95)	1,052 (93)
Weeks gestation at birth		
< 37	339 (5)	96 (8)
≥ 37	7,163 (95)	1,037 (92)
Mother’s age at delivery (years)		
< 20	934 (12)	111 (10)
20–24.9	3,274 (44)	523 (46)
25–29.9	2,120 (28)	336 (30)
30–34.9	856 (11)	125 (11)
≥ 35	313 (4)	38 (3)
No. of full-term live births		
1	2,813 (37)	524 (46)
2	2,306 (31)	433 (38)
≥ 3	2,348 (31)	176 (16)
Mother’s ethnicity		
Czech/other	6,640 (89)	1,041 (92)
Roma	856 (11)	92 (8)
Mother’s education		
Did not complete primary school	120 (2)	11 (1)
Primary school	1,587 (21)	190 (17)
Some secondary school	3,205 (43)	497 (44)
Secondary with leaving exam	2,092 (28)	343 (30)
University degree/student	4,400 (59)	68 (6)
No. of cigarettes/day smoked by mother before pregnancy		
None	4,584 (61)	725 (64)
1 to 10	1,593 (21)	233 (21)
11 to 20	1,033 (14)	120 (11)
≥ 21	130 (2)	12 (1)

a Some categories do not sum to 7,502 or 1,133 due to missing data and/or rounding.

**Table 3:** Bronchitis^a^ rates and rate ratios, from birth through 23 months of age.

Covariate	No. of events	Child months	Rate per child month	Crude RR (95% CI)	Multivariate^b^ RR (95% CI)
Overall	1,429	26,214	0.05		
District					
Prachatice	648	11,190	0.06	Reference	Reference
Teplice	781	15,024	0.05	0.90 (0.81–1.00)	1.05 (0.82–1.34)
Mother’s age (years)					
< 20	849	14,619	0.06	1.11 (1.00–1.24)	1.08 (0.89–1.32)
20–29.9	557	10,692	0.05	Reference	Reference
≥ 30	23	902	0.03	0.49 (0.32–0.74)	0.48 (0.33–0.71)
Mother’s education					
Low	340	4,566	0.07	1.88 (1.62–2.17)	1.77 (1.31–2.40)
Medium	675	11,455	0.06	1.49 (1.31–1.69)	1.33 (1.06–1.67)
High	382	9,640	0.04	Reference	Reference
Unknown	32	553	0.06	1.46 (1.02–2.09)	—c
Mother or other adults smoke					
No	432	10,564	0.041	Reference	Reference
Yes	997	15,649	0.064	1.56 (1.39–1.74)	1.35 (1.09–1.67)
Child’s sex					
Male	834	13,226	0.06	1.38 (1.24–1.53)	1.38 (1.13–1.68)
Female	595	12,988	0.05	Reference	Reference
Child’s age (months)					
0–3	82	3,372	0.02	0.44 (0.35–0.55)	0.60 (0.41–0.88)
> 3–6	191	3,231	0.06	1.07 (0.91–1.25)	1.49 (1.16–1.92)
> 6–12	433	6,529	0.07	1.20 (1.07–1.35)	1.28 (1.09–1.49)
> 12–24	723	13,082	0.06	Reference	Reference
Season					
Winter	528	6,289	0.084	3.21 (2.70–3.80)	1.31 (0.93–1.84)
Spring	339	6,597	0.051	1.96 (1.64–2.35)	1.61 (1.25–2.08)
Summer	177	6,758	0.026	Reference	Reference
Fall	385	6,570	0.059	2.24 (1.87–2.67)	1.61 (1.24–2.09)
Day of the week					
Monday	364	3,746	0.097	1.59 (1.41–1.80)	1.70 (1.47–1.97)
Tuesday–Friday	916	14,987	0.061	Reference	Reference
Saturday–Sunday	149	7,481	0.020	0.20 (0.27–0.39)	0.30 (0.24–0.38)
Fuel for heating and/or cooking					
Coal	200	2,634	0.076	1.43 (1.23–1.66)	1.35 (1.01–1.79)
Gas	559	11,535	0.048	0.91 (0.78–1.07)	0.91 (0.70–1.19)
Electricity and distant heat	1,066	20,041	0.053	Reference	Reference
Breast-feeding category					
Current	232	5,984	0.039	0.59 (0.49–0.71)	0.64 (0.44–0.93)
0–3 months ago	146	2,749	0.053	0.80 (0.65–1.00)	0.78 (0.53–1.17)
> 3 months ago	844	14,370	0.059	0.89 (0.76–1.04)	0.92 (0.65–1.31)
Never	201	3044	0.066	1.00	1.00
Unknown	6	67	0.089	1.35 (0.60–3.05)	—c
No. of other children ≤ 14 years of age in the home					
≥ 1	954	15,449	0.062	1.40 (1.25–1.56)	1.30 (1.04–1.63)
0	473	10,694	0.044	1.00	1.00
Calendar year (per year)					1.03 (0.95–1.11)
14-day average temperature (per degree C)					0.97 (0.95–0.98)
30-day average PAH^d^					
High^d^	494	5,691	0.087	2.52 (2.22–2.87)	
Medium	492	7,646	0.064	1.87 (1.65–2.13)	
Low	443	12,877	0.034	Reference	
Continuous^e^					1.29 (1.07–1.54)
30-day average PM_2.5_^d^					
High*d*	85	799	0.106	2.26 (1.81–2.82)	
Medium	454	6,525	0.070	1.48 (1.32–1.65)	
Low	890	18,890	0.047	Reference	
Continuous^e^					1.30 (1.08–1.58)

a ICD-10 codes for bronchitis are J20 and J21.

b Adjustment is for all other variables in multivariate model, except that only one of the air pollutants was included at a time (model shown is for PAHs, but covariates showed virtually identical results when PM_2.5_ was substituted for PAHs). Models were also adjusted for repeated measures within each child, as well as for sampling design.

c Categories with unknown values were excluded.

d High PAHs defined as > 100 ng/m^3^; medium as 40–100 ng/m^3^. High PM_2.5_ defined as > 50 μg/m^3^; medium as 25–50 μg/m^3^.

e Per increment of 2 SDs (100 ng/m^3^ PAHs; 25 μg/m^3^ PM_2.5_)

**Table 4:** Bronchitis^a^ rates and rate ratios, from 2 to 4.5 years of age.

Covariate	No. of events	Child months	Rate per child month	Crude RR (95% CI)	Multivariate^b^ RR (95% CI)
Overall	1,052	27,367	0.038		
District					
Prachatice	509	11,554	0.044	Reference	Reference
Teplice	543	15,813	0.034	0.78 (0.69–0.88)	0.75 (0.59–0.96)
Mother or other adults smoke					
No	391	10,706	0.037	Reference	Reference
Yes	661	16,661	0.040	1.09 (0.96–1.23)	1.26 (1.04–1.53)
Child’s sex					
Male	585	13,602	0.043	1.27 (1.12–1.43)	1.26 (1.03–1.53)
Female	467	13,765	0.034	Reference	Reference
Child’s age (months)					
24–36	571	12,836	0.044	1.33 (1.11–1.58)	
> 36–42	154	5,369	0.029	0.86 (0.69–1.07)	
> 42–48	166	4,360	0.038	1.14 (0.91–1.41)	
> 48	161	4,803	0.034	Reference	
Child’s age in months (continuous)					0.94 (0.94–0.95)
Season					
Winter	392	6,372	0.062	3.52 (2.87–4.32)	
Spring	227	7,168	0.032	1.81 (1.45–2.26)	
Summer	121	6,928	0.017	Reference	
Fall	312	6,899	0.045	2.59 (2.10–3.19)	
Day of the week					
Monday	272	3,917	0.069	1.53 (1.33–1.75)	1.54 (1.31–1.82)
Tuesday–Friday	711	15,625	0.046	Reference	Reference
Saturday–Sunday	69	7,825	0.009	0.19 (0.15–0.25)	0.23 (0.17–0.32)
Fuel for heating and/or cooking					
Coal	111	2,599	0.043	1.00 (0.82–1.22)	1.13 (0.82–1.56)
Gas	383	11,715	0.033	0.76 (0.67–0.87)	0.86 (0.68–1.08)
Electricity and dist heat	558	13,053	0.043	Reference	Reference
Breast-feeding ever					
No	131	3,189	0.041	Reference	
Yes	920	24,123	0.038	0.93 (0.77–1.11)	
Unknown	1	55	0.018	0.44 (0.06–3.17)	
Child currently attending daycare or preschool/kindergarten					
No	675	19,724	0.034	Reference	Reference
Yes	373	7,432	0.050	1.47 (1.29–1.66)	2.12 (1.70–2.64)
Unknown	4	211	0.019	0.55 (0.21–1.48)	—c
No. of other children ≤ 14 years of age in the home					
1 or more	672	16,547	0.041	1.16 (1.02–1.31)	1.22 (1.00–1.48)
0	380	10,820	0.035	Reference	Reference
Calendar year					1.16 (1.08–1.26)
14-day average temperature (per degree C)					0.95 (0.94–0.97)
30-day average PAH^d^					
High^d^	213	3,029	0.070	2.26 (1.93–2.65)	
Medium	285	6,527	0.044	1.40 (1.20–1.64)	
Low	554	17,811	0.031	Reference	
Continuous^e^					1.56 (1.22–2.00)
30-day average PM 2.5^d^					
High^d^	12	98	0.122	3.66 (2.07–6.48)	
Medium	346	6,466	0.054	1.60 (1.41–1.82)	
Low	694	20,803	0.033	Reference	
Continuous^e^					1.23 (0.94–1.62)

a ICD-10 codes for bronchitis are J20 and J21.

b Adjustment is for all other variables in multivariate model, except that only one of the air pollutants was included at a time (model shown is for PAHs, but covariates showed virtually identical results when PM_2.5_ was substituted for PAHs). Models also adjusted for repeated measures within each child, as well as for sampling design.

c Categories with unknown values were excluded.

d High PAHs defined as > 100 ng/m^3^; medium as 40–100 ng/m^3^. High PM_2.5_ defined as > 50 μg/m^3^; medium as 25–50 μg/m^3^.

e Per increment of 2 SDs (100 ng/m^3^ PAHs; 25μg/m^3^ PM_2.5_).
